# Modulating myosin restores muscle function in a mouse model of nemaline myopathy

**DOI:** 10.1002/ana.24619

**Published:** 2016-03-22

**Authors:** Johan Lindqvist, Yotam Levy, Alisha Pati‐Alam, Edna C. Hardeman, Paul Gregorevic, Julien Ochala

**Affiliations:** ^1^ Department of NeuroscienceUppsala UniversityUppsalaSweden; ^2^ Centre of Human and Aerospace Physiological Sciences, Faculty of Life Sciences & MedicineKing's College LondonLondonUnited Kingdom; ^3^ Neuromuscular and Regenerative Medicine Unit, School of Medical SciencesUniversity of New South WalesSydneyNew South WalesAustralia; ^4^ Baker IDI Heart and Diabetes InstituteMelbourneVictoriaAustralia; ^5^ Department of Biochemistry and Molecular BiologyMonash UniversityMelbourneVictoriaAustralia; ^6^ Department of PhysiologyUniversity of MelbourneMelbourneVictoriaAustralia; ^7^ Department of NeurologyUniversity of Washington School of MedicineSeattleWA

## Abstract

**Objective:**

Nemaline myopathy, one of the most common congenital myopathies, is associated with mutations in various genes including *ACTA1*. This disease is also characterized by various forms/degrees of muscle weakness, with most cases being severe and resulting in death in infancy. Recent findings have provided valuable insight into the underlying pathophysiological mechanisms. Mutations in *ACTA1* directly disrupt binding interactions between actin and myosin, and consequently the intrinsic force‐generating capacity of muscle fibers. *ACTA1* mutations are also associated with variations in myofiber size, the mechanisms of which have been unclear. In the present study, we sought to test the hypotheses that the compromised functional and morphological attributes of skeletal muscles bearing *ACTA1* mutations (1) would be directly due to the inefficient actomyosin complex and (2) could be restored by manipulating myosin expression.

**Methods:**

We used a knockin mouse model expressing the *ACTA1* His40Tyr actin mutation found in human patients. We then performed in vivo intramuscular injections of recombinant adeno‐associated viral vectors harboring a myosin transgene known to facilitate muscle contraction.

**Results:**

We observed that in the presence of the transgene, the intrinsic force‐generating capacity was restored and myofiber size was normal.

**Interpretation:**

This demonstrates a direct link between disrupted attachment of myosin molecules to actin monomers and muscle fiber atrophy. These data also suggest that further therapeutic interventions should primarily target myosin dysfunction to alleviate the pathology of *ACTA1*‐related nemaline myopathy. Ann Neurol 2016;79:717–725

Nemaline myopathy is one of the most common forms of congenital myopathy, with a prevalence that differs between studies (overall <20% of all congenital myopathies).^1^, ^2^, ^3^ Seven genes related to this disease encode proteins involved in muscle contraction: *ACTA1*, *NEB*, *TPM3*, *TPM2*, *CFL2, TNNT1*, and *LMOD3*.^1^, ^4^, ^5^ *KBTBD13, KLHL40*, and *KLHL41* genes have also been associated with nemaline myopathy but encode proteins contributing to transcriptional regulation and degradation.^1^

*ACTA1* mutations are often missense, changing 1 DNA nucleotide and leading to the replacement of 1 amino acid in the skeletal muscle α‐actin protein. For most cases, these subtle substitutions induce severe frailty and early mortality.^4^ Nevertheless, various degrees of limb, masticatory, and respiratory muscle weakness exist.^4^ Recent studies using human biopsy tissue and mouse models have helped to identify some of the cellular and molecular events that underlie muscle weakness.^6^, ^7^, ^8^ Mutations in *ACTA1* often produce actin mutants that behave as “poison proteins” and modify the binding interaction between myosin and actin filaments. This disruption of interaction between myosin and actin monomers diminishes the establishment of cross‐bridges in the strong binding state, thus preventing optimal force‐generating capacity at the level of the myofiber.^6^, ^7^, ^8^

Despite these crucial advances in our understanding of the pathophysiology of *ACTA1*‐related nemaline myopathy, to date, no cure exists. One reason may lie in the underappreciation of muscle fiber smallness as another major contributor of weakness associated with *ACTA1* mutations.^4^ This variation in myofiber size is defined, in most patients, by a significant atrophy or hypotrophy of type I muscle cells.^4^ As the mechanisms that contribute to this have not been elucidated, we sought to investigate this aspect of disease by using a knockin transgenic mouse model expressing a single residue substitution in skeletal muscle α‐actin (His40Tyr), as a model of the human condition.^7^, ^9^ The majority of myofibers (type I as well as type II) from this animal model are atrophic or hypotrophic.^9^

We initially hypothesized that the defective myosin binding to actin filaments and subsequent aberrant intrinsic force‐generating capacity observed in the presence of His40Tyr would directly limit the force to be sensed, altering mechanosensitive pathways responsible for protein turnover, promoting myofiber atrophy or hypotrophy, and ultimately resulting in severe weakness and early death (60% of the mice die by 13 weeks of age).^7^, ^10^ To test this hypothesis, we attempted to restore the actomyosin interface in the transgenic mouse model by directly modulating the myosin light chains (MyLCs). Each myosin molecule is composed of 2 regulatory and 2 essential/alkali MyLCs.^11^ Essential/alkali MyLCs have C‐terminal regions involved in the coupling of adenosine triphosphate (ATP) hydrolysis/myosin arm rotation and N‐terminal domains capable of directly interacting with actin.^11^ Thus, different MyLC isoforms with distinct amino acids in their N‐ or C‐termini are able to promote or limit force production. For instance, MyLC1_a/emb_, which is encoded by the *MYL4* gene and only present in the heart and skeletal muscles of embryos, increases the force‐generating capacity of myosin molecules.^11^ From that, we hypothesized that inducing expression of MyLC1_a/emb_ in mature skeletal myofibers via transgene delivery (with recombinant adeno‐associated virus [rAAV] vectors containing the *MYL4* gene) would (1) improve the interactions between myosin and actin filaments in wild‐type mice, (2) restore the contractile function in the knockin transgenic mouse model, and (3) prevent atrophy or hypotrophy by ameliorating the mechanosensitive pathways responsible for protein turnover.

We found that vector‐mediated expression of *MYL4* significantly ameliorated the atrophy and associated deficiencies in contractile performance of muscle fibers in transgenic mice. By using MyLC1_a/emb_, we did not intend to directly correct for the His40Tyr mutation, but rather to compensate for it by specifically improving myosin cross‐bridge function. Thus, our findings suggest that *MYL4*‐directed interventions could offer promising therapeutic potential for the treatment of neuromuscular disorders where an impaired myosin binding is observed.

## Materials and Methods

### Animals

Six wild‐type male mice as well as 6 age‐ and gender‐matched transgenic mice expressing the His40Tyr amino acid substitution in skeletal α‐actin were included in the analyses. For a complete description of the mice, please see Nguyen et al.^9^

### Production of rAAV Vectors (rAAV6)

A cDNA construct encoding myosin light chain 4 (*MYL4*, GenScript) was cloned into an AAV expression plasmid consisting of a cytomegalovirus promoter/enhancer and an SV40 poly‐A region flanked by AAV2 terminal repeats using standard cloning techniques. Transfection of these plasmids with the pDGM6 packaging plasmid into HEK293 cells generated type 6 pseudotyped viral vectors that were harvested and purified as described previously.^12^ Briefly, HEK293 cells were plated at a density of 3.2 to 3.8 × 10^6^ cells on a 10cm culture dish, 8 to 16 hours prior to transfection with 10μg of a vector‐genome–containing plasmid and 20μg of the packaging/helper plasmid pDGM6, by means of the calcium phosphate precipitate method to generate pseudotype 6 vectors. Seventy‐two hours after transfection, the media and cells were collected and homogenized through a microfluidizer (Microfluidics, Westwood, MA) prior to 0.22μm clarification (Millipore, Billerica, MA). The vector was purified from the clarified lysate by affinity chromatography over a HiTrap heparin column (GE Healthcare, Little Chalfont, UK), and ultracentrifuged overnight prior to resuspension in sterile physiological Ringer solution. The purified vector preparation was titered with a customized sequence‐specific quantitative polymerase chain reaction–based reaction (Applied Biosystems, Foster City, CA) as described previously.^13^

### In Vivo Intramuscular Injections

Four‐week‐old mice (5 wild‐type and 6 transgenic males) were deeply anesthetized with isoflurane, and ∼1 × 10^10^ vector genomes of rAAV6 containing the *MYL4* gene (referred to as rAAV6:MYL4) were injected in 30μl of Hank balanced salt solution directly into the anterior compartment of the hindlimb, which is occupied by the tibialis anterior muscle. Control injections of the contralateral limb used a vector lacking a functional gene (referred to as rAAV6:MCS). Four weeks after the injections, animals were killed by cervical dislocation under deep isoflurane sedation and tibialis anterior muscles were dissected. All procedures involving animal care, welfare, and handling were performed according to institutional guidelines and were reviewed and approved by the Uppsala Local Ethical Committee on Animal Research.

### Muscle Dissection and Preparation

Upon surgical excision, harvested tibialis anterior muscles were separated into 2 portions. One piece was directly frozen in liquid nitrogen‐chilled propane and stored at −80°C. The other piece was placed in relaxing solution at 4°C. Bundles of approximately 50 fibers were dissected free and then tied with surgical silk to glass capillary tubes at just taught lengths. Prepared muscle bundles were subsequently treated with skinning solution (relaxing solution containing glycerol; 50:50 vol/vol) for 24 hours at 4°C, after which they were transferred to −20°C. In addition, the muscle bundles were treated with sucrose, a cryoprotectant, within 1 to 2 weeks for long‐term storage.^14^ They were detached from the capillary tubes and snap frozen in liquid nitrogen‐chilled propane and stored at −80°C.

### Functional Measurements

Bundles of prepared muscle fibers were desucrosed and transferred to a relaxing solution, and single fibers were dissected. To measure the functional properties, myofibers 1 to 2mm long were affixed between connectors leading to a force transducer (model 400A; Aurora Scientific, Aurora, Ontario, Canada) and a lever arm system (model 308B, Aurora Scientific).^15^, ^16^ The 2 extremities of the fiber segment were tightly attached to the connectors as previously described.^15^ The apparatus was mounted on the stage of an inverted microscope (model IX70; Olympus, Tokyo, Japan). The sarcomere length was set to 2.50 to 2.60μm (optimal mouse sarcomere length where the force production is the highest) and controlled during the experiment using a high‐speed video analysis system (model 901A HVSL, Aurora Scientific). The diameter of the muscle fiber segment mounted between the connectors was measured through a microscope at a magnification of ×320 with an image analysis system prior to the mechanical experiments. Myofiber depth was measured by recording the vertical displacement of the microscope nosepiece while focusing on the top and bottom surfaces of the fiber. The focusing control of the microscope was used as a micrometer. Muscle fiber cross‐sectional area (CSA) was calculated from the diameter and depth, assuming an elliptical circumference, and was corrected for the 20% swelling that is known to occur during skinning.^15^ Diameter and depth were measured at 3 different locations along the length of each fiber segment, and the mean was considered representative of cell dimensions. As previously described in detail,^17^, ^18^, ^19^ mechanical experiments were conducted at 15°C and included force measurements (normalized to CSA) after various length steps at saturating [Ca^2+^] (pCa = 4.50). Step changes in fiber length were imposed and allowed stiffness measurements (releases of 0.15, 0.3, and 0.5% of fiber length and stretches of the same amplitudes). Stiffness was defined as the slope of the linear regression of the relationship between the peak force response and the length change.

Relaxing and activating solutions contained (in millimolars) 4 Mg‐ATP, 1 free Mg^2+^, 20 imidazole, 7 ethyleneglycoltetraacetic acid (EGTA), 14.5 creatine phosphate, and KCl to adjust the ionic strength to 180mM and pH to 7.0. The preactivating solution was identical to the relaxing solution except that the EGTA concentration was reduced to 0.5mM. The concentrations of free Ca^2+^ were 10^−9^M (relaxing and preactivating solutions) and 10^−4.50^M (activating solution), expressed as pCa (ie, −log10 [Ca^2+^]). The rigor activating solution was similar to the regular activating solution except that MgATP and creatine phosphate were absent.

After the functional measurements, each myofiber was placed in urea buffer (120g urea, 38g thiourea, 70ml H_2_0, 25g mixed bed resin, 2.89g dithiothreitol, 1.51g Trizma base, 7.5g sodium dodecyl sulfate [SDS], 0.004% bromophenol blue) in a plastic microcentrifuge tube and stored at −80°C. The myosin heavy chain (MyHC) isoform composition was subsequently determined by 6% SDS–polyacrylamide gel electrophoresis.^15^, ^16^ The acrylamide concentration was 4% (wt/vol) in the stacking gel and 6% in the running gel, and the gel matrix included 30% glycerol. Sample loads were kept small (equivalent to approximately 0.05mm of fiber segment) to improve the resolution of the MyHC bands (types I, IIa, IIx, and IIb). Electrophoresis was performed at 120V for 24 hours with a Tris–glycine electrode buffer (pH 8.3) at 15°C (SE 600 vertical slab gel unit; Hoefer Scientific Instruments, San Francisco, CA). The gels were silver‐stained and subsequently scanned in a soft laser densitometer (Molecular Dynamics, Sunnyvale, CA) with a high spatial resolution (50μm pixel spacing) and 4,096 optical density levels. It should be mentioned that as no significant differences were seen between the various MyHC isoforms, all the functional data were deemed eligible for pooling together.

### Cytochemistry and Immunocytochemistry

Ten‐micrometer cross‐sections were cut perpendicular to the longitudinal axis of the muscle fibers with a cryostat (2800 Frigocut E; Reichert‐Jung, Vienna, Austria) for determination of morphological characteristics with standard staining protocols and of MyLC1_a/emb_ expression.

Primary antibody against MyLC_1a/emb_ from rabbit was diluted 1:100,^20^ and antirabbit secondary antibody (1:500 dilution) conjugated with Alexa Flour 488 was detected by fluorescence microscopy using a Zeiss (Oberkochen, Germany) LSM 510 Meta (PlanNeofluar ×20/0.5 objective). In addition, cross‐sections were processed using standard histological and histochemical procedures. Pictures were captured with a microscope‐mounted camera (DPII camera; Olympus), and calculations of fiber size were determined using imaging software (Compix Simple PCI 6; Compix, Irvine, CA).

### Immunoblotting

Markers of potentially activated signaling pathways were evaluated using Western blots. Four micrograms of total protein were loaded per lane into the wells of a 4% (wt/vol) acrylamide stacking gel with 12% (wt/vol) running gel; the gel matrix included 10% glycerol. Electrophoresis was performed at 120V for approximately 90 minutes, and the gels were immediately transferred to polyvinylidene fluoride membranes (Immobilon FL, Millipore). Membranes were blocked with 2% ECL Prime (GE Healthcare) blocking agent for 1 hour and then incubated with mTOR (#2972; Cell Signaling Technology, Danvers, MA), p‐mTOR (#2974, Cell Signaling Technology), PI3K (#4257, Cell Signaling Technology), p‐Akt (#9271, Cell Signaling Technology), and Akt (#9272, Cell Signaling Technology) primary antibodies. Primary antibodies against α‐actinin (A7732; Sigma‐Aldrich, St Louis, MO) were used for normalization. All primary antibodies were raised in rabbit except for α‐actinin, which was raised in mouse. After incubation with secondary antibodies conjugated with horseradish peroxidase against rabbit (NA934, Amersham GE Healthcare) or rat (NA931, Amersham GE Healthcare), blots were developed with ECL Prime detection kit according to the manufacturer's instruction (Amersham GE Healthcare). The immunoblots were sequentially quantified using the chemiluminescence setting in an imaging system (Odyssey Fc Infrared Imaging System; LI‐COR, Lincoln, NE).

### Statistical Analysis

Data are reported as means ± standard error of the mean. Sigma Stat software (Jandel Scientific, San Rafael, CA) and Excel (Microsoft, Redmond, WA) were used to generate descriptive statistics. Differences between the groups were determined by 1‐way or 2‐way analyses of variance depending on the data (*p*‐value set at 0.05).

## Results and Discussion

### Myofiber Atrophy Appeared at 8 Weeks of Age in Transgenic Mice

**Figure 1:**
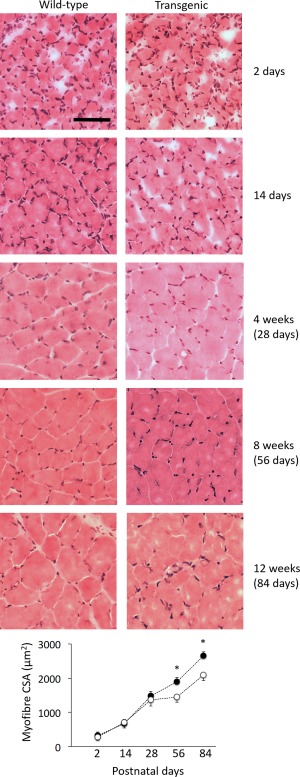
Tibialis anterior muscles of transgenic mice exhibit myofiber atrophy at week 8. This figure displays representative muscle cross‐sections taken at different time points and stained with hematoxylin and eosin. These sections did not exhibit any major cores or vacuoles but display minor artifacts of cryodamage during processing. Values in the graphs are mean ± standard error of the mean. *Statistically significant at *p* < 0.05. The scale bar represents 50μm. CSA = cross‐sectional area. [Color figure can be viewed in the online issue, which is available at www.annalsofneurology.org.]

To determine whether muscle cells were atrophic or hypotrophic as a consequence of *ACTA1* mutation, we evaluated the CSA of fibers at different age points in transgenic mice (2 days, 14 days, 4 weeks, 8 weeks, and 12 weeks after birth). Smaller fibers were observed toward the end of the episode of postnatal maturation (ie, 8 and 12 weeks, *p* < 0.05; Fig 1), which favors atrophic mechanisms rather than hypotrophy. These could originate from decreased protein synthesis or from increased protein degradation. Time‐resolved analyses in humans are warranted to determine whether this holds true for patients or whether impaired processes of muscle growth occur.

Having identified a time course for the development of muscle fiber atrophy associated with *ACTA1* mutation, we sought to investigate whether features of atrophy could be ameliorated by altering actin–myosin interaction. For subsequent experiments, we chose to use 4‐week old wild‐type and transgenic mice (just before the atrophy onset) that were sampled for examination at 8 weeks of age.

### rAAV6:MYL4 Hyperactivated Myosin Molecules in Wild‐Type Mice

**Figure 2:**
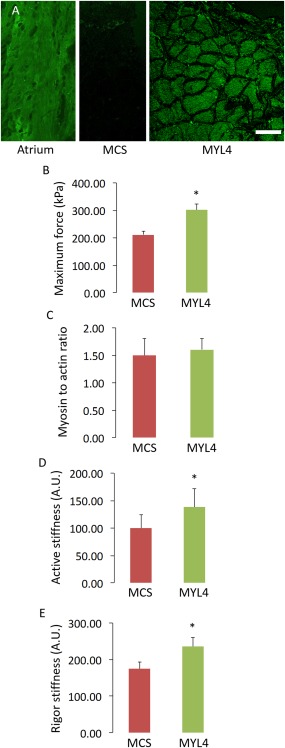
Incorporation of the transgene in wild‐type mice improves the myofiber function. (A) Typical muscle cross‐sections from the atrium or tibialis anterior muscles from wild‐type animals. The tibialis anterior muscles were injected with ∼1 × 10^10^ vector genomes of rAAV6 containing either the *MYL4* gene (referred to as MYL4) or a vector lacking a functional gene (referred to as MCS). These cross‐sections were stained for MyLC_1a/emb._ The scale bar represents 50μm. (B–E) B, D, and E are graphs depicting the myofiber mechanics, whereas C summarizes the myosin to actin content ratio in these myofibers. Values in the graphs are mean ± standard error of the mean. *Statistically significant at *p* < 0.05. [Color figure can be viewed in the online issue, which is available at www.annalsofneurology.org.]

Recombinant AAV vectors have been demonstrated to achieve highly efficient and long‐lasting transduction of mammalian skeletal musculature.^21^ We used rAAV6 vectors to transduce the muscles of mice with transgene constructs harboring *MYL4*. As shown in Figure 2, in wild‐type mice, injections of rAAV6:MYL4 elicited strong expression of MyLC1_a/emb_, as detected by immunofluorescent labeling. Treatment of muscles with rAAV6:MYL4 led to a marked (∼50%) increase in the steady‐state isometric maximal force‐producing capacity of myofibers when compared with muscles receiving the rAAV6:MCS control vector (*p* < 0.05). Such increase may originate from either a greater amount of myosin cross‐bridges in the strong binding state and/or a higher force per individual actomyosin interaction. To distinguish between these two possibilities, we performed stiffness measurements on isolated muscle fiber segments. As active and rigor stiffness were both greater with rAAV6:MYL4 when compared with rAAV6:MCS (*p* < 0.05), and as the myosin to actin content ratio was not affected, we attributed the increased force production to a greater strain per individual myosin cross‐bridge, which may be due to a change in the rigidity of the neck region of the myosin motor.^22^

### rAAV6:MYL4 Restored the Intrinsic Force‐Generating Capacity and Myofiber Size in Transgenic Mice

**Figure 3:**
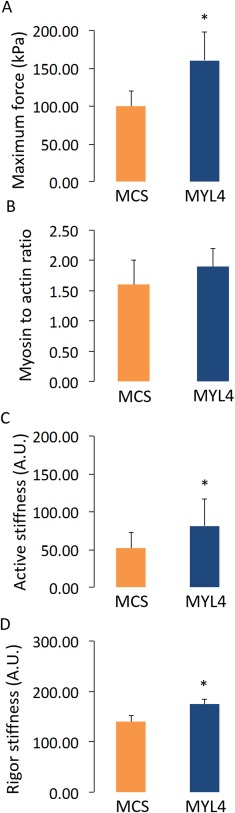
The myosin transgene alleviates the myofiber dysfunction in transgenic mice. A, C, and D relate to the myofiber contractile function in tibialis anterior muscles that were injected with ∼1 × 10^10^ vector genomes of rAAV6 containing either the *MYL4* gene (referred to as MYL4) or a vector lacking a functional gene (referred to as MCS), whereas C reports the myosin to actin content ratio in the myofibers. Values in the graphs are mean ± standard error of the mean. *Statistically significant at *p* < 0.05. [Color figure can be viewed in the online issue, which is available at www.annalsofneurology.org.]

**Figure 4:**
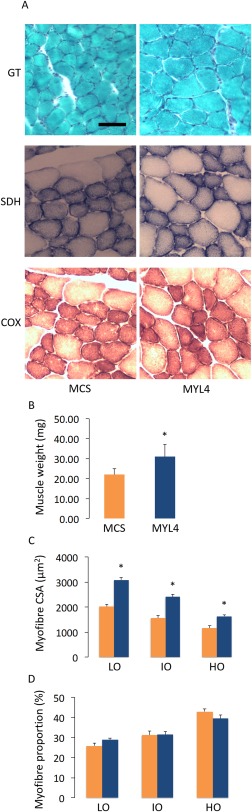
The myosin transgene restores muscle mass in transgenic mice. (A) Typical muscle cross‐sections from the tibialis anterior muscles from transgenic mice. The tibialis anterior muscles were injected with ∼1 × 10^10^ vector genomes of rAAV6 containing either the *MYL4* gene (referred to as MYL4) or a vector lacking a functional gene (referred to as MCS) and were stained with Gomori trichrome (GT), succinic dehydrogenase (SDH), or cytochrome oxidase (COX). The scale bar represents 50μm. (B) The muscle weights. (C) Myofiber cross‐sectional areas (CSA) are classified according to their oxidative capacity (LO = low; IO = intermediate; HO = high). (D) The proportion of myofibers having LO, IO, or HO. Values in the graphs are mean ± standard error of the mean. *Statistically significant at *p* < 0.05. [Color figure can be viewed in the online issue, which is available at www.annalsofneurology.org.]

**Figure 5:**
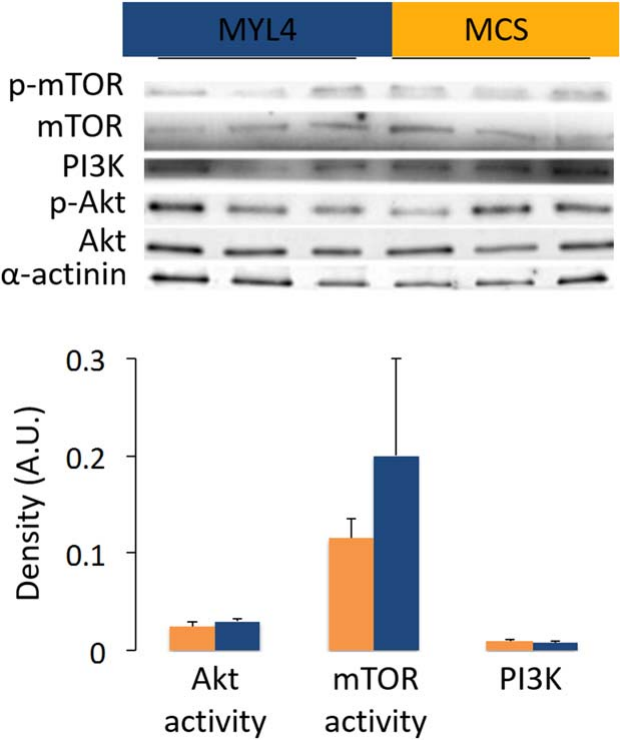
The functional beneficial effects of the myosin transgene do not involve the PI3K‐Akt‐mTOR signaling in transgenic mice. This figure shows typical Western blots of the tibialis anterior muscles from transgenic mice. These muscles were injected with ∼1 × 10^10^ vector genomes of rAAV6 containing either the *MYL4* gene (referred to as MYL4) or a vector lacking a functional gene (referred to as MCS). The p‐Akt to Akt and p‐mTOR to mTOR ratios were calculated and represented the Akt and mTOR activities, respectively. Values in the graphs are mean ± standard error of the mean. [Color figure can be viewed in the online issue, which is available at www.annalsofneurology.org.]

Consistent with the observed increases in force‐producing capacity achieved in wild‐type mice, administration of rAAV6:MYL4 to the muscles of *ACTA1* mutant mice also improved the steady‐state isometric maximal force production, and active and rigor stiffness of myofibers when compared with muscles receiving rAAV6:MCS (*p* < 0.05; Fig 3). Moreover, we noticed that the presence of MyLC1_a/emb_ stimulated an increase in muscle weight and myofiber CSA (*p* < 0.05; Fig 4B, C) without any alteration in the proportional composition of MyHC isoforms/oxidative capacity (see Fig 4D). The regulation of muscle fiber size is influenced by the balance between rates of protein degradation and synthesis.^23^ Hence, to gain insight into the underlying causes of the increased CSA with rAAV6:MYL4, we sought to determine whether increased muscle fiber size was a function of altered signaling via the Akt‐mTOR pathway, a major determinant of protein turnover in muscle.^24^ We did not detect differences in the expression or activation of the upstream element PI3‐kinase, or in the activation of Akt and mTOR as determined from the p‐Akt to Akt and p‐mTOR to mTOR ratios (Fig 5). These findings indicate that other signaling processes, such as other mechanosensitive pathways, may be responsible for the increase in myofiber CSA.^25^ Based on one of our previous studies on muscle disuse/unloading,^26^ one may speculate that, in the presence of His40Tyr, the limited myosin binding and force production lead to a partial mechanical silencing that would trigger a complex cascade of molecular events that could activate FoxO and key muscle‐specific E3 ligases such as MuRF‐1 and atrogin‐1/MAFbx. The incorporation of MyLC1_a/emb_ would be predicted to exert a protective effect upon mechanosensing and downstream signaling, which could limit FoxO‐related detrimental effects and thus favor myofiber growth.

### MYL4 as a Potential Future Therapy for Nemaline Myopathy

Interestingly, the results of the present study also demonstrate that a molecular intervention designed to promote expression of MyLC1_a/emb_ in skeletal musculature can ameliorate features of pathology associated with *ACTA1*‐related nemaline myopathy. This particular MyLC isoform is not commonly expressed in adult skeletal muscles, but strongly supports myosin cross‐bridge binding to actin filaments. Other molecular interventions have been proposed as a means to target the contractility of fibers by sensitizing the troponin–tropomyosin regulatory complex to Ca^2+^ or by directly stimulating the myosin molecules, albeit with limited promise in the context of nemaline myopathy to date. For instance, a pharmacological drug (CK‐2017357) containing a troponin activator has been tested.^27^ By selectively binding to the troponin complex, CK‐2017357 slows the rate of Ca^2+^ release from troponin C and facilitates myosin activation. Despite this obvious beneficial effect, CK‐2017357 suffers a major limitation in that it dramatically slows the rate of Ca^2+^ dissociation from troponin C, impairing the relaxation process.^27^ Another compound, which directly attaches to myosin molecules (omecamtiv mecarbil, formerly CK‐1827452), has successfully shown inotropic effects.^28^ However, omecamtiv mecarbil specifically binds to cardiac myosin molecules, and thus would not exert efficacy as a therapy for skeletal muscles. By comparison, the *MYL4*‐based intervention described here demonstrates a capacity to considerably enhance the intrinsic contractile capacity of *ACTA1*‐mutant muscle fibers, and ameliorate fiber atrophy associated with this disease model. As nemaline myopathy related to defects in other genes encoding for contractile proteins (ie, *NEB*, *TPM3*, *TPM2*, *CFL2, TNNT1*, and *LMOD3*) is often characterized by depressed myosin cross‐bridge function, one may suggest that the beneficial compensatory mechanisms related to the incorporation of MyLC1_a/emb_ in skeletal muscles may be applicable to these other genetic forms. Accordingly, our intervention strategy represents a novel and efficacious approach to preventing and/or treating muscle pathology in the setting of nemaline myopathy.

### Conclusion

The results reported here demonstrate that administration of recombinant viral vectors carrying a *MYL4* construct (1) enhances the interactions between myosin and actin filaments in wild‐type mice, with beneficial effects for contractile capacity; and (2) prevents muscle atrophy and enhances the intrinsic force‐generating capacity in *ACTA1*‐mutant mice. Given the potential for rAAV vectors to achieve widespread and long‐lasting dissemination of transgenes to skeletal musculature in vivo, we contend that further examination of this intervention strategy is warranted to determine the potential for development as a novel therapy for nemaline myopathy and other neuromuscular disorder‐related defects of interaction within the contractile architecture of muscle fibers. Important questions to address in future studies include (1) evaluating the long‐term effects of this intervention strategy; (2) determining whether it is possible to ameliorate disease features in limb, masticatory, and respiratory muscle via systemic vector administration; and (3) establishing whether *MYL4*‐based gene therapy is beneficial in mouse models of nemaline myopathy carrying other gene defects such as *NEB* and *TPM3*.

## Author Contributions

J.O. and P.G. contributed to conception and design of the studies. All authors contributed to the acquisition and analysis of data, and to drafting the text and preparing the figures.

## Potential Conflicts of Interest

Nothing to report.
